# Morbidity and Mortality in Patients Undergoing Mitral Valve Replacement at a Cardiovascular Surgery Referral Service: a Retrospective Analysis

**DOI:** 10.21470/1678-9741-2019-0440

**Published:** 2021

**Authors:** Júlia Lasserre Moreira, Pedro Henrique Andrade Araújo Salvatore Barletta, José Augusto Baucia

**Affiliations:** 1 Faculdade de Medicina da Bahia, Universidade Federal da Bahia, Salvador, Bahia, Brazil.; 2 Department of Anesthesiology and Surgery, Universidade Federal da Bahia, Salvador, Bahia, Brazil.

**Keywords:** Aged, Mitral Valve Insufficiency, Cardiovascular Diseases, Stroke Volume, Endocarditis, Hemoglobins, Rheumatic Diseases, Medical Records

## Abstract

**Introduction:**

We aimed to identify predictors of morbidity and mortality in patients undergoing isolated mitral valve replacement.

**Methods:**

This is a retrospective cohort study with 164 patients who underwent isolated mitral valve replacement at a referral hospital for cardiovascular diseases, which were performed from January 2011 to December 2016. Data were obtained from medical records, including preoperative, intraoperative, and postoperative information. Statistical analysis was performed to calculate odds ratio (OR), unpaired Student's *t*-test, and binary logistic regression. *P*-values < 0.05 were considered significant.

**Results:**

A total of 69.5% (n=114) of the patients had a diagnosis of rheumatic disease prior to surgery. Mortality rate was 6.7% (n=11). The most observed complication was the occurrence of postoperative arrhythmias (19.5%). On average, patients remained 5.34 days in the intensive care unit. There was a statistically significant enhanced risk of death among patients with previous diagnosis of endocarditis (OR 5.22, 95% confidence interval [CI] 1,368-19,915; *P*=0.008), reduced ejection fraction (EF) (< 50%) (OR 9.46, 95% CI 2,61-34,35; *P*<0.001), and mitral regurgitation (MR) (OR 7.7, 95% CI 1.576-37.545; *P*=0.004). Patients who died were older than those who survived surgery (*P*<0.001) and had lower preoperative serum hemoglobin levels (*P*=0.018). Logistic regression showed age and reduced EF at preoperative evaluation as predictors of death.

**Conclusion:**

Older age, reduced serum hemoglobin levels, preoperative diagnosis of endocarditis, reduced EF, and MR were associated with postoperative mortality. Age and reduced EF were predictors of death.

**Table:** 

Abbreviations, acronyms & symbols				
**AF**	**= Atrial fibrillation**		**Ht**	**= Hematocrit**
**BMI**	**= Body mass index**		**ICU**	**= Intensive care unit**
**CI**	**= Confidence interval**		**INR**	**= International Normalized Ratio**
**CPB**	**= Cardiopulmonary bypass**		**LV**	**= Left ventricular**
**Cr**	**= Creatinine**		**MR**	**= Mitral regurgitation**
**EF**	**= Ejection fraction**		**NYHA**	**= New York Heart Association**
**EuroSCORE**	**= European System for Cardiac Operative Risk Evaluation**		**OR**	**= Odds ratio**
			**PT**	**=Prothrombin time**
**Hb**	**=Hemoglobin**		**Ur**	**=Urea**

## INTRODUCTION

Mitral valve disease is the most common valvular heart disorder^[1]^. Both mitral stenosis and regurgitation lead to pulmonary hypertension and right heart failure. Mitral valve replacement is currently one of the most common treatments for rheumatic fever, the main underlying etiology in developing countries^[1]^.

Approximately 33 million people live with rheumatic heart disease, leading to around 275.000 annual deaths^[2]^. Most data on demographics, morbidity, and mortality in the literature, as well as factors associated with patient outcomes, are reported by studies performed in developed countries, with starkly different social and medical realities than those found in developing countries^[2]^. In most high-income countries, rheumatic heart disease has not been a public health problem for decades^[2]^.

Mitral valve operations are the fastest growing surgical intervention reported by the Society of Thoracic Surgeons Adult Cardiac Surgery Database analysis^[3]^. Currently, rates of mitral valve replacement are decreasing, as valve repair is preferable whenever possible^[1,4]^. Operative mortality associated with isolated mitral valve replacement is reported in between 4% and 7% of patients^[5]^. Thromboembolism, the most common postoperative complication, has been reported to occur at a rate of 1.5% to 2.0% per patient-year^[5]^.

Successful outcomes are determined by the correct choice of technique, patient risk factors, the use of appropriate prosthetics, proper anticoagulant management, and efficient postoperative clinical follow-up^[5]^. Recent studies have shown that variables, including advanced disease, age, and debilitating comorbidities, are associated with worse mitral valve surgical outcome^[5]^.

The relative high rate of morbimortality in patients submitted to heart valve surgery has led to numerous prospective and retrospective studies attempting to estimate its risk. These studies aimed to detect patients who face higher surgical risk through the assessment of clinical and demographic characteristics in order to reduce surgical mortality and morbidity, as well as the cost of care^[6]^.

Several preoperative and postoperative factors may increase patient risk related to valve replacement. Surgical experience has shown that preoperative factors, such as the use of intra-aortic balloon pumping, renal failure on dialysis, and endocarditis, as well as postoperative factors, including sepsis, cardiopulmonary bypass (CPB) time, and valve reoperation, among others, all contribute to in-hospital mortality^[7]^. Nevertheless, the contribution of other variables to short- or long-term mortality rates, *e.g*. atrial fibrillation (AF), continues to be debated by some authors^[8,9]^.

This study aimed to identify preoperative, intraoperative, and postoperative risk factors related to morbidity and mortality in a Brazilian population of patients who underwent isolated mitral valve replacement, as well as to investigate sociodemographic characteristics and clinical profiles, in order to obtain pertinent information regarding the current scenario in Brazil.

## METHODS

### Study Design and Population

The present retrospective cohort study analyzed the medical records of 164 patients who underwent isolated mitral valve replacement at Ana Nery Hospital, a cardiovascular disease referral center located in the city of Salvador (Bahia, Brazil).

The primary outcome was death during hospitalization for isolated mitral valve replacement surgery, while the secondary outcome was the incidence of morbidity in the postoperative period.

### Inclusion Criteria

Patients who underwent isolated mitral valve replacement between 2011 and 2016, regardless of age, valve lesion etiology, or type of prosthesis, were included.

### Exclusion Criteria

Any patients who underwent mitral valve replacement in association with any other surgical procedure were excluded, as were those whose information was missing or whose medical records were unavailable.

### Variables

Clinical and laboratory data regarding the preoperative, intraoperative, and postoperative periods were collected using medical records and through the application of a standardized form. Table 1 lists all variables and outcomes investigated.

**Table 1:** Variables and outcomes.

1. Age	
2. Gender	
3. Height	
4. Weight	
5. Body mass index	
6. NYHA functional classification	
7. Preoperative comorbidities:	a. Stroke
	b. Diabetes mellitus
	c. Rheumatic fever
	d. Hypertension
	e. Endocarditis
	f. Asthma
	g. Smoking
	h. Coronary artery disease
	i. Atrial fibrillation
	j. Chronic obstructive pulmonary disease
	k. Previous valve operation
8. Urea	
9. Creatinine	
10. Hb (g/dl)	
11. Ht (%)	
12. Prothrombin time	
13. INR	
14. Rhythm of electrocardiogram	
15. Echocardiographic measurements	
16. EuroSCORE	
17. Urgency or elective surgery	
18. Prosthesis model	
19. Prosthesis size	
20. Mitral calcification	
21. Duration of extracorporeal circulation	
22. Aortic clamping time	
23. Length of ICU stay	
24. Outcomes	a. Acute myocardial infarction
	b Stroke
	c. Respiratory tract infection
	d. Arrhythmia
	e. Reoperation
	f. Cardiac tamponade
	g. Hb (mg/dl)
	h. Mediastinitis
	i. Sepsis
	j Endocarditis
	k Other
	l. Death

EuroSCORE=European System for Cardiac Operative Risk Evaluation; Hb=hemoglobin; Ht=hematocrit; ICU=intensive care unit; INR=International Normalized Ratio; NYHA=New York Heart Association

### Clinical and Demographic Characteristics

Of 207 patients, 43 were excluded due to lack of information in medical records, leaving 164 for analysis. Females predominated (n=104; 63.4%), with a median age of 44 (±14.43) years. The median weight and body mass index values were 62 (± 13.19) kg and 23.33 (±4.66) kg/m^2^, respectively. Forty-six (28.05%) patients had no record of use of any medications, while 118 (71.95%) were on drug therapy, mostly diuretics (n=92; 56.1%) and beta-blockers (n=81; 49.4%). Thirty (18.3%) patients had received penicillin.

Preoperative evaluations indicated that 93.3% of the patients presented at least one comorbidity, and most (n=114; 69.5%) had been diagnosed with rheumatic heart disease prior to surgery (Table 2).

**Table 2:** Preoperative comorbidities.

Comorbidities		Frequency
Rheumatic disease		114 (69.5%)
Hypertension		63 (38.4%)
Atrial fibrillation		62 (37.8%)
Smoking or former smoking		28 (17.1%)
Stroke	Once	23 (14%)
	More than once	3 (1.8%)
Endocarditis		20 (12.2%)
Diabetes mellitus		16 (9.8%)
Asthma		7 (4.3%)
Coronary artery disease		5 (3%)
Chronic obstructive pulmonary disease		1 (0.6%)
NYHA functional class	1	4 (2.7%)
	2	75 (50.3%)
	3	56 (37.6%)
	4	14 (9.4%)

NYHA=New York Heart Association

Previous mitral repair had been performed in 27 (16.5%) patients, one patient (0.6%) had undergone double valve replacement, and 32 (19.5%) had been submitted to prior mitral valve replacement. The median European System for Cardiac Operative Risk Evaluation score, or EuroSCORE, was 2.55 (±6.32). Table 3 lists the preoperative exam findings prior to the mitral valve surgery in question.

**Table 3:** Preoperative laboratory results.

Exams	Median (standard deviation)	Frequencies
Hemoglobin (g/dL)	12.4 (±1.99)	-
Hematocrit (%)	37 (±28.18)	-
Creatinine (mg/dL)	1 (±0.91)	-
Urea (mg/dL)	30 (±24.36)	-
International Normalized Ratio	1.29 (±1.30)	-
Prothrombin time (%)	70.5 (±23.93)	-
Left atrium (mm)	52 (±14.50)	-
Aorta (mm)	29 (±5.20)	-
LV systolic diameter (mm)	33 (±8.74)	-
LV diastolic diameter (mm)	53 (±9.51)	-
Posterior wall thickness (mm)	8 (±1.72)	-
Interventricular septum (mm)	8 (±1.74)	-
LV ejection fraction (%)	65 (±11.8)	-
Pulmonary artery systolic pressure (mmHg)	48 (±18.05)	-
Calcification	-	51 (31.3%)
Paravalvular leak	-	33 (20.2%)

LV=left ventricular

Most patients presented sinus rhythm on the preoperative electrocardiogram (n=76; 46.3%), with no other changes described. Sixty-two patients (37.8%) had AF, two had (1.2%) atrial flutter, four (2.4%) had bradycardia, and three (1.8%) had tachycardia. Four patients (2.4%) presented other cardiac alterations.

The most commonly reported valve dysfunction was the presence of mixed mitral valve lesions (n=62; 37.8%), followed by mitral valve regurgitation (n=61; 37.2%) and stenosis (n=27; 16.4%). In patients who previously received a valve prosthesis, the following dysfunctions were observed: regurgitation (n=8; 4.9%), stenosis (n=4; 2.4%), and mixed lesions (n=1; 0.6%). The most common associated valve lesion was tricuspid regurgitation, present in 74 (45.1%) patients.

A total of 161 (98.2%) patients underwent surgery on an elective basis, while just three (1.8%) were classified as urgent. The most commonly used prosthesis was biological (n=115; 70.1%) and the most common size was 29 mm (n=70; 42.7%), followed by 31 mm (n=61; 37.2%) and 27 mm (n=12; 7.3%). Papillary muscle and chordae tendineae were preserved in all patients with left ventricular (LV) dysfunction. Prophylactic anticoagulation therapy against postoperative thrombosis was begun immediately after drain removal.

### Statistical Analysis

Statistical analysis was performed using the IBM Corp. Released 2015, IBM SPSS Statistics for Windows, Version 23.0, Armonk, NY: IBM Corp. software. Categorical variables are presented as absolute and relative frequencies, while continuous variables are presented as means, medians, and standard deviations. Associations between categorical variables were investigated through odds ratio (OR) analysis. The Kolmogorov-Smirnov test was used to certify the data distribution normality. Means were compared using the unpaired Student's *t*-test. Binary logistic regression was employed to identify possible predictors of postoperative death using Nagelkerke's coefficient of determination (R²). *P*-values < 0.05 were considered statistically significant.

### Ethical Considerations

This study was approved by the Ethics in Research Committee of the Ana Nery Hospital (protocol no. 336.981, approved on 7/19/13). In accordance with Resolution No. 466/2012 of the National Council of Ethics in research involving human beings, this study protocol was registered on the Plataforma Brasil website (CAAE number 14268813.5.0000.0045). The authors signed a term of commitment regarding the use of data contained in the patients' medical records. The standardized form used to collect patient information was approved by the local institutional review board.

## RESULTS

The overall mortality rate was 6.7% (n=11), with the leading cause of deaths being infection (n=4; 36.4%), followed by cardiogenic shock (n=2; 18.2%) and stroke (n=2; 18.2%), and one death registered due to chagasic cardiomyopathy (9%). In two cases, no specific cause of death was recorded. The most common postoperative complication was arrhythmia (n=32; 19.5%), followed by respiratory tract infection (n=24; 14.6%) and renal failure (n=13; 7.9%) (Table 4). The most common arrhythmia was AF (n=25; 78.1%), followed by supraventricular tachycardia (n=3; 9.4%) and ventricular tachycardia (n=2; 6.25%). One case each of atrial flutter, non-specific bradycardia, and complete heart blockage were reported (3.1% for each). On average, patients remained in the intensive care unit (ICU) for 5.34 (± 4.31) days, with a median length of stay of four days.

**Table 4:** Postoperative outcomes.

Outcome	Frequencies
Arrhythmias	32 (19.5%)
Respiratory tract infection	24 (14.6%)
Renal insufficiency	13 (7.9%)
Death	11 (6.7%)
Pericardial effusion	9 (5.5%)
Surgical wound infection	8 (4.9%)
Sepsis	7 (4.3%)
Pleural effusion	7 (4.3%)
Mediastinitis	5 (3%)
Reoperation	5 (3%)
Urinary tract infection	4 (2.4%)
Cardiac tamponade	2 (1.2%)
Endocarditis	2 (1.2%)
Subcutaneous emphysema	2 (1.2%)
Stroke	2 (1.2%)
Pneumothorax	2 (1.2%)
Acute heart failure	1 (0.6%)
Pulmonary thromboembolism	1 (0.6%)

Comparing the mean age of patients who died after surgery with those who survived, a statistically significant difference was observed (62.0 *vs*. 43.4 years; *P*<0.001), indicating that older age was associated with the primary outcome. Patients who died presented lower mean preoperative serum hemoglobin levels (11.02 g/dl *vs*. 12.49 g/dl; *P*=0.018).

A previous diagnosis of rheumatic disease was associated with a decreased risk of postoperative death (OR 0.035, 95% confidence interval [CI] 0.004-0.278; *P*<0.001). However, a significantly lower age was found in these patients than in those who did not receive this diagnosis (40.2 years *vs*. 54.6 years; *P*<0.001).

The presence of arrhythmias in the preoperative period was not found to be associated with death following valve replacement (OR 1.5, 95% CI 0.417-5.708; *P*=0.513) (Table 5). The same was observed for patients with a previous diagnosis of coronary artery disease (OR 3,70, 95% CI 0.377-36.281; *P*=0.23), hypertension (OR 3.0, 95% CI 0.841-10.703; *P*=0.078), and stroke (OR 1.171, CI 95 % 0.241-5.829; *P*=0.834). Since no diabetics died, it was impossible to establish associations with diabetes mellitus and the primary outcome. The New York Heart Association (NYHA) functional classes III/IV were also not associated with higher mortality (OR 1.442, 95% CI 0.372-5.598; *P*=0.595).

**Table 5:** Odds ratio values representative of risk-related outcomes.

Variable	n (%)	Deaths	Odds ratio	95% confidence interval	*P*-value
Women	103 (62.8%)	5 (4.9%)	0.459	0.134 - 1.575	0.207
Obesity (BMI > 30 kg/m^2^)	16 (9.7%)	1 (6.25%)	1,133	0.130 - 9.857	0.91
Arrhythmias	75 (45.73%)	6 (8%)	1,543	0.417-5.708	0.513
NYHA functional class III/IV	70 (42.68%)	5 (7.1%)	1,442	0.372 - 5.598	0.595
Stroke	26 (15.85%)	2 (7.7%)	1.185	0.241 - 5.829	0.834
Rheumatic disease	114 (69.51%)	1 (0.9%)	0.035	0.004-0.278	<0.001
Chronic obstructive pulmonary disease	1 (0.6%)	0	-	-	0.787
Coronary artery disease	5 (3%)	1 (20%)	3.7	0.377 - 36.281	0.23
Diabetes	16 (9.8%)	0	-	-	0.257
Hypertension	63 (38.4%)	7 (11.7%)	3	0.841 - 10.703	0.078
Asthma	7 (4.3%)	0	-	-	0.467
Smoking	28 (17.1%)	2 (7.1%)	1.077	0.220 - 5.277	0.927
Atrial fibrillation	62 (37.8%)	4 (6.5%)	0.926	0.260 - 3.302	0.906
Endocarditis	19 (11.58%)	4 (21.2%)	5.219	1.368 - 19.915	0.008
Previous valve surgery	59 (36%)	1 (1.7%)	0.162	0.020 - 1.299	0.053
Urgent surgery	3 (1.8%)	0	-	-	0.654
Mitral calcification	51 (31.1%)	1 (2%)	0.227	0.028 - 1.839	0.131
LV ejection fraction < 50%	23 (14%)	6 (26.1%)	9.459	2.605 - 34.35	<0.001
Mitral regurgitation	60 (36.6%)	8 (13.33%)	7.692	1.576 - 37.545	0.004
Mitral stenosis	27 (16.46%)	0	-	-	0.114
Mixed mitral lesion	62 (37.8%)	2 (3.2%)	0.383	0.079 - 1.867	0.220
Lesions in other valves	132 (80.49%)	10 (7.6%)	2.295	0.282 - 18.672	0.425
Paravalvular leak	12 (7.3%)	0	-	-	0.356

BMI=body mass index; LV=left ventricular; NYHA=New York Heart Association

A preoperative diagnosis of endocarditis was associated with a greater risk of death (OR 5.219, 95% CI 1.368-19.915; *P*=0.008), regardless of patient age. An ejection fraction < 50% was also associated with postoperative mortality (OR 9.459, 95% CI 2.605-34.350; *P*<0.001).

Mitral regurgitation (MR) was found to be associated with the primary outcome (OR 7.692, 95% CI 1.576-37.545; *P*=0.004). No other types of associated valvular heart disease were found to be related with evolution to death.

A longer period of intraoperative myocardial anoxia (57.94 min *vs*. 79.09 min; *P*=0.008) was observed in patients who died postoperatively, as well as a longer duration of cardiopulmonary bypass (76.14 min *vs*. 101.82 min; *P*=0.004), in comparison to those who survived (Table 6).

**Table 6:** Students' t-test for numerical variables.

Variable	Median±standard deviation	Non-survivors	Survivors	*P*-value
Age	44±14.43	62±10.1	43.43±13.9	<0.001
BMI	23.3±4.66	24.13±5.48	23.92±4.63	0.919
Days in ICU	4±4.31	10.44±11.22	5.05±3.385	0.739
Preoperative Hb (g/dl)	12.4±2	11.02±2.27	12.49±1.94	0.018
Ht (%)	37±28.18	33.9±6.95	39.18±29.20	0.108
Cr (mg/dL)	1±091	1.13±0.72	1.16±0.93	0.894
Ur (mg/dL)	30±24.37	66.92±5.45	34±18.93	0.078
INR	1.29±1,3	1.49±0.59	1.55±1.34	0.781
PT (%)	70.5±24	72.45±25.91	70±23.93	0.764
Left atrium (mm)	52±14.5	55.22±15.64	53±14.48	0.692
Aorta (mm)	29±5.2	32.41±6.39	29.2±5.08	0.174
LV diastolic diameter (mm)	53±9.5	55.4±11	53.06±9.44	0.549
LV systolic diameter (mm)	33±8.7	35.4±10.18	34.5±8.68	0.813
Interventricular septum (mm)	8±1.74	7.5±3.33	8.29±1.62	0.54
Systolic pulmonary artery pressure (mmHg)	48±18.1	56.62±16.02	49.45±18.15	0.257
LV ejection fraction (%)	65±11.8	53.62±17.24	63.33±11.1	0.093
Posterior wall thickness (mm)	8±1.72	7.42±3.55	8.28±1.59	0.553
EuroSCORE	2.5±6.32	6.4±7.37	4.46±6.26	0.411
Postoperative Hb (g/dl)	9.58±1.79	9±1.87	9.53±1.79	0.399
Cardiopulmonary bypass length	70±29.01	101.82±81.16	76.14±20.6	0.004
Myocardial anoxia	55±25.64	79.1±74.48	57.94±17.39	0.008

BMI=body mass index; Cr=creatinine; EuroSCORE=European System for Cardiac Operative Risk Evaluation; Hb=hemoglobin; Ht=hematocrit; ICU=intensive care unit; INR=International Normalized Ratio; LV=left ventricular; PT=prothrombin time; Ur=urea

Considering multivariate analysis by binary logistic regression, a significant model was obtained that included age and reduced ejection fraction (< 50%) as variables (χ² (2) = 25,871, *P*<0.001; R² Nagelkerke = 0.377) (Table 7). Age (OR 1.117, 95% CI 1.045-1.193) and reduced ejection fraction (OR 6.444, 95% CI 1.572-26.746) were predictors of postoperative death. The same did not happen for the presence of preoperative endocarditis, MR, and serum hemoglobin levels.

**Table 7:** Binary Logistic Regression Model - Death.

$R^{2}=0.377\left(\mathrm{Nagel}\ker\mathrm{ke}\right)$ $X^{2}\left(2\right)=25.871,P<0.001$		B	Significance	OR	95% CI to OR	
					Inferior	Superior
Variables	Age	0.110 (±0.034)	*P*=0.001	1.117	1.045	1.193
	Low EF	1.869 (±0.723)	*P*=0.010	6.484	1.572	26.746
	Constant	-9.119 (±2.113)	*P*<0.001	-	-	-

CI=confidence interval; EF=ejection fraction; OR=odds ratio; R^2^=determination coefficient; χ^2^=chi square

## DISCUSSION

The predominance (63.4%; n=44) of female patients in this study is compatible with the female majority in Brazilian population. The Global Rheumatic Heart Disease Registry, or the REMEDY study^[2]^, and Remenyi, ElGuindy, Smith, Yacoub, and Holmes^[4]^ also showed a predominance of this sex among the total affected by rheumatic disease, whether in developed countries or not. Most studies on mitral surgery have a mostly female population^[10-17]^.

The mean age of patients undergoing valve replacement differs according to the population. Studies in high-income countries have valve degeneration secondary to aging or ischemic valve injury as the main etiology leading to replacement. Hence, they can show a slightly higher average age^[10,14]^. Studies carried out on samples with rheumatic being the most prevalent etiology show lower averages, as well as that found in the present study^[16,17]^.

There is no consensus regarding the predictors of valve surgery mortality, and their identification varies widely according to the population studied and study method. Age, for example, has already been identified as an important predictor numerous times^[4,7,8,12-14,18-21]^, in agreement with what was found here. On the other hand, a number of other studies did not show its relationship with worse outcome^[11]^ or even identified the inverse relationship^[22]^.

As already established, rheumatic etiology of valve disease remains frequent in Brazil and in other underdeveloped or in developing countries. This study corroborates this information as it alerts to the need for control and prevention of disease progression. There is still no evidence associating rheumatic disease with changes in postoperative outcomes. Khan et al.^[22]^ observed a relationship between high blood anti-streptolysin O levels and operative mortality. However, these are markers of active rheumatic fever and not its sequel, rheumatic disease.

In this study, the previous diagnosis of rheumatic fever was associated with better postoperative outcome, showing itself as a protective factor. However, patients with a previous diagnosis of this disease had a statistically significant lower age than patients without such diagnosis, which probably interfered with the result found.

The type of valve lesion has not yet been associated with worse outcomes, contrasting with the association of MR with higher mortality found here. A study by Khan et al.^[22]^ failed to establish relationship between the type of injury and higher mortality. Likewise, it is not yet well established which associated valve lesions imply the highest surgical risk, although tricuspid regurgitation has already been identified as an independent risk factor for death and morbidity^[12,23]^.

Among the preoperative laboratory variables, only the low hemoglobin value was associated with worse outcome. This value is probably related to the patients' previous comorbidities, which consequently contribute to a worse outcome, although none of them individually was associated with it. In the literature, the only variable already associated is the high postoperative creatinine value^[20,22]^.

In the present study, no association was found between symptom severity (demonstrated by NYHA functional class) and mortality, corroborating the results presented by Khan et al.^[22]^. However, this finding shows a contrast when compared to previous studies, such as Appelbaum, Kouchoukos, Blackstone, and Kirklin^[11]^; Bourguignon et al.^[18]^; Bourguignon et al.^[17]^; Rahimtoola^[21]^; and Lam et al.^[13]^ that showed lower mortality in the NYHA class I/II subgroup compared to III/IV. However, none of the studies cited had a population identical to this one.

Among the preoperative comorbidities, coronary artery disease is the most frequently identified as a predictor of mortality. Numerous studies have already shown its association with worse outcome^[12-14,19]^. Other comorbidities, such as stroke, hypertension, chronic obstructive pulmonary disease, and chronic renal failures have also been identified^[13,24]^. In the present study, only endocarditis was associated, as described by Toumpoulis, Chamogeorgakis, Angouras, Swistel, Anagnostopoulos, and Rokkas^[7]^ and Rankin et al.^[19]^.

AF is known to be an important and prevalent arrhythmia that accompanies mitral injury. However, its association with postoperative mortality is still contradictory. In some studies^[4,8,9,12,21,22]^ this arrhythmia has already been identified as an independent predictor factor. In the present study, however, such association was not found, a situation also verified by the study by Lim et al.^[25]^.

As described before^[4,8,13,19,20,21]^, patients with reduced ejection fraction (< 50%) presented worse outcomes. However, other preoperative echocardiographic variables were not associated with higher mortality, as found in previous studies^[8,13]^.

Duration of CPB and time of anoxia were longer among patients who died, as described by Appelbaum, Kouchoukos, Blackstone, and Kirklin^[11]^ and Rahimtoola^[21]^. This finding is consistent since myocardial distress is greater as longer the duration of CPB is.

We found a predominance of biological prostheses and recent studies indicate an increase in use of bioprosthetic mitral valve^[15]^. There was no association of any of the characteristics of the prostheses with higher mortality. Some studies agree with that finding^[15]^, while some identify bioprosthesis as a predictor of worse outcome^[13]^.

We found here a high average ICU stay (5.34 days), even though most patients were NYHA functional class III/IV. This finding can be explained by the fact that the routine of ICU stay in the service is three days, added to the occurrence of complications such as infections, acute renal failure, and reoperations, as seen in Table 4.

The hospital mortality found in this study (6.7%) was similar to that found in most other studies on valve replacement^[10,11,14]^, being within the normal range (4 to 7%)^[5]^. The causes of death vary among studies. Appelbaum, Kouchoukos, Blackstone, and Kirklin^[11]^ found all deaths related to a low cardiac performance (low cardiac output), just like Rankin et al.^[19]^ showed a majority of deaths (60,3%) due to cardiac causes. On the other hand, Eguchi et al.^[9]^ observed that all deaths were related to infection. Most studies show infection, cardiac causes, and neurological events (which are the main causes in this study) as frequent causes of death in valvular procedures^[9,11,12,14,19,20]^.

### Limitations

The present study has some limitations. It is a retrospective study at a single center. It was based on data obtained from medical records and problems in its filling may have interfered with the results. The number of individuals was limited and may not have been enough to reach statistical significance in some situations. For all those reasons, population inferences should be analyzed with caution. It is also good to remember that Brazil is a very heterogeneous country, and the study was made in a single region, where rheumatic disease is still endemic and responsible for most of the cases referred to surgery for mitral disease. These results may not be inferred for other regions.

## CONCLUSION

Advanced age, reduced preoperative serum hemoglobin levels, previous endocarditis, reduced LV ejection fraction, and MR were associated with a greater risk of mortality. Rheumatic disease was associated with better surgical outcomes. Age and reduced ejection fraction at preoperative evaluation were predictors of death after isolated mitral valve replacement.

**Table:** 

Authors' roles & responsibilities	
JLM	Substantial contributions to the conception or design of the work; or the acquisition, analysis, or interpretation of data for the work; drafting the work or revising it critically for important intellectual content; agreement to be accountable for all aspects of the work in ensuring that questions related to the accuracy or integrity of any part of the work are appropriately investigated and resolved; final approval of the version to be published
PHAASB	Substantial contributions to the conception or design of the work; or the acquisition, analysis, or interpretation of data for the work; drafting the work or revising it critically for important intellectual content; final approval of the version to be published
JAB	Substantial contributions to the conception or design of the work; or the acquisition, analysis, or interpretation of data for the work; final approval of the version to be published
